# Determining the Metabolic Footprints of Hydrocarbon Degradation Using Multivariate Analysis

**DOI:** 10.1371/journal.pone.0081910

**Published:** 2013-11-25

**Authors:** Renee. J. Smith, Thomas C. Jeffries, Eric M. Adetutu, Peter G. Fairweather, James G. Mitchell

**Affiliations:** 1 School of Biological Sciences, Flinders University, Adelaide, South Australia, Australia; 2 Plant Functional Biology and Climate Change Cluster, University of Technology Sydney, Sydney, New South Wales, Australia; Dowling College, United States of America

## Abstract

The functional dynamics of microbial communities are largely responsible for the clean-up of hydrocarbons in the environment. However, knowledge of the distinguishing functional genes, known as the metabolic footprint, present in hydrocarbon-impacted sites is still scarcely understood. Here, we conducted several multivariate analyses to characterise the metabolic footprints present in a variety of hydrocarbon-impacted and non-impacted sediments. Non-metric multi-dimensional scaling (NMDS) and canonical analysis of principal coordinates (CAP) showed a clear distinction between the two groups. A high relative abundance of genes associated with cofactors, virulence, phages and fatty acids were present in the non-impacted sediments, accounting for 45.7 % of the overall dissimilarity. In the hydrocarbon-impacted sites, a high relative abundance of genes associated with iron acquisition and metabolism, dormancy and sporulation, motility, metabolism of aromatic compounds and cell signalling were observed, accounting for 22.3 % of the overall dissimilarity. These results suggest a major shift in functionality has occurred with pathways essential to the degradation of hydrocarbons becoming overrepresented at the expense of other, less essential metabolisms.

## Introduction

Ecosystem functioning is highly dependent on microbial communities[1–3]. These communities are largely defined by a set of metabolic pathways, and are generally thought to be habitat specific [4], providing a link between the biology of a given community and the surrounding environment [5]. Environmental change can lead to a major shift in the structure and function of the inhabiting microbial consortia [6–8]. Physiological adaptations of microbes have been shown to be highly specific, allowing for the discrimination between chemical stressors [9]. The identification of defining metabolic pathways of a given ecosystem, known as metabolic footprints, allows for a greater understanding on how the microbial consortia are adapting and responding to environmental change [10,11]. 

Microorganisms are highly responsive to environmental stress, due to a variety of evolutionary adaptations and physiological mechanisms [12]. The innate ability of microbes to respond and adapt to the world around them means they are often used as biological indicators [13], and subsequently for bioremediation [14]. Many studies have investigated the use of specific microbial taxa as biological indicators [15–18]; however, the key distinguishing metabolisms associated with hydrocarbon contamination are less well characterized than the taxa. Previous reports have shown that metagenomes are highly predictive of metabolic potential within an ecosystem [3]. Furthermore, previous studies have shown that microbial communities often respond at a metabolic level before any disturbance is seen at the taxonomic level [17]. Therefore, to gain comprehensive insight into an ecosystem’s functional response to environmental change, the underlying metabolic footprints should be elucidated.

Metabolic footprints is a term used to describe an ensemble of biological pathways that typically occur with a combination of environmental variables [10,19]. Due to the great diversity of metabolic pathways present within microbial communities, the determination of a metabolic footprint requires the use of multivariate analysis. A recent study by Gianoulis et al. [10] used multivariate canonical correlation analysis to describe the metabolic footprints associated with different marine environments. These metabolic footprints were thought to arise from differences in evolutionary strategies required to cope with unique environmental variables [10]. Similarly, Dinsdale et al. [4] used canonical correlations to discriminate between 9 discrete ecosystems. 

Typically metabolic footprint studies employ constrained ordination tools, such as canonical discriminant analysis (CDA) and principal component analysis (PCA) [4,20,21], to explore the metabolic footprints of an environment. However, these methods are restricted in that PCA cannot be performed on datasets containing more variables (e.g. taxa/metabolic processes) than observations (samples), and CDA should be performed on a dataset where there are at least three times as many observations than variables [22]. This limitation results in the need to reduce the number of variables prior to analysis [20]. Microbial communities, however, comprise intricate networks whereby a large number of individuals and metabolic processes are important in the overall ecosystems functioning [23]. Thus, the community as a whole should be considered when categorising a given environment.

Canonical analysis of principal coordinates (CAP) is thus a constrained multivariate analysis, that uses both ordination and discrimination function techniques, but, unlike CDA and PCA it better allows for the characterisation of whole communities as it is not limited by observation size due to its testing via permutation [24]. Furthermore, canonical analysis of principal coordinates (CAP) is highly constrained to the hypothesis, allowing for discriminations to be made in strongly correlated variables, such as functional processes [25]. PERMANOVA on the other hand is affected by other variables that may be present within a given dataset, making it less able to detect differences in less abundant functional subsystems [46]. CAP analysis has been used in several studies to determine how microbial communities respond to various environmental conditions [25–28]; however, to date, it has not been employed to generate and explore metabolic footprints for impacted environments. Thus, we sought to construct a metabolic profile of microbial communities responding to various forms of environmental impacts, in order to generate metabolic footprints using CAP.

The long-lasting toxicity of xenobiotics makes their metabolism by microbial communities widely studied [29]. Petroleum hydrocarbons are a common target for bioremediation because they are widespread and persistent [7,30–33]. While the taxa and environmental conditions for optimal degradation of hydrocarbons are well established [34–37], the effectiveness of a natural community to bioremediate is less well understood [38]. 

Advances in metagenomic technologies have allowed for the direct sequencing of environmental microbial communities [39], greatly increasing our potential to understand the metabolic processes being undertaken by the indigenous microbial communities. A recent study by Yergeau et al. [40] used metagenomic sequencing technologies to characterise the structure and function of an active soil microbial community in a hydrocarbon contaminated Arctic region. However, this study primarily focused on the taxa present, and not the defining metabolic activities associated with hydrocarbon contamination. Thus, knowledge about the distinguishing functional genes present in hydrocarbon contaminated environments is still lacking. 

The aim of the present study was to compare hydrocarbon-impacted sites to non-impacted sites, and provide insight into the key metabolic functions present following hydrocarbon impact, thus elucidating the metabolic footprints for hydrocarbon contamination. The robustness of these metabolic footprints were assessed with the inclusion of metagenomes from a variety of geographical locations and substrate types, experiencing different contamination events.

## Materials and Methods

### Data Collection

To determine the functionality of microbial communities inhabiting hydrocarbon-impacted and non-impacted environments, publicly available datasets were chosen from the MetaGenomics Rapid Annotation using Subsystem Technology (MG-RAST) pipeline version 3.0 [41]. Due to constraints in the database, a total of 4 datasets were used to represent hydrocarbon-impacted environments, while 5 datasets were used for non-impacted environments (Table S1 in File S1). BLASTX was performed on all datasets, with a minimum alignments length of 50 bp and an E-value cut-off of E<1×10^-5^ [4], to identify hits to the subsystems database. 

### Data Analysis

To statistically investigate the differences between metagenomes from hydrocarbon-impacted sites to metagenomes from non-impacted sites, heatmaps were generated containing the relative proportion of hits to the subsystem database in MG-RAST. Heatmaps had been standardized and scaled to account for differences in sequencing effort and read lengths. Statistical analysis was conducted on square-root transformed data to reduce the impact of dominant metabolisms using the software package PRIMER 6 for Windows (Version 6.1.13, Primer-E, Plymouth) [42]. To generate a robust set of metabolic footprints, the generalized cellular functions, termed level 1, and the subsystem, termed level 2 hierarchical classifications were used to determine the overall differences in metabolic potential [4,10]. 

To determine whether there was any loss of information between the levels of resolution for metabolism, the program RELATE in the PRIMER package was used to calculate the Spearman rank correlation between hierarchical levels 1 and 2 [43]. Differences in the overall metabolic potential between hydrocarbon-impacted and non-impacted sediments were analysed using the PERMANOVA+ version 1.0.3 3 add-on to PRIMER [44,45]. Non-metric Multi-Dimensional scaling (NMDS) of Bray-Curtis similarities was performed as an unconstrained ordination method to graphically visualise multivariate patterns in the metabolic processes associated with hydrocarbon-impacted or non-impacted sediment metagenomes. Metagenomes were further analysed using canonical analysis of principal coordinates (CAP) on the sum of squared canonical correlations as a constrained ordination and discrimination method, to determine whether there was any significant difference between metabolic processes according to hydrocarbon impact. The *a priori* hypothesis that the metabolisms between the two groups were different was tested in CAP [45] by obtaining a P value using 9999 permutations. Based on RELATE results, CAP ordinations were generated using hierarchy level 1 for metabolism.

Where significant differences were found using CAP, the percent contribution of each metabolism to the separation between the hydrocarbon-impacted and non-impacted samples were assessed using similarity percentage (SIMPER) analysis [43]. The resulting top 90 percent of all metabolisms were used to determine the shifts in metabolic potential between the groups. To determine those metabolisms that were consistently contributing most to the overall dissimilarity between the hydrocarbon-impacted and non-impacted groups, the ratio of the average dissimilarity to standard deviation was used. A dissimilarity/standard deviation (Diss/SD) ratio of greater than 1.4 was used to indicate key discriminating metabolisms [46]. 

To assess the robustness of the metabolic footprints generated using this method, three common forms of environmental impact (agricultural, hydrocarbon and wastewater) from a diverse range of geographical locations and substrate types were compared (Table S1 in File S1). Firstly, heatmaps were generated as above and the square-root transformed data was analysed using Primer 6 for windows. The CAP on the sum of squared canonical correlations [44] was performed to graphically illustrate the multivariate patterns of metabolism associated with these impacted environments. Significant trends in metabolic processes at each site were determined using the sum of squared canonical correlations. The *a priori* hypothesis that the metabolisms among the four groups were different was tested using 9999 permutations. Where statistically significant differences were shown using CAP analysis, similarity percentage (SIMPER) analysis [43] was conducted as above to determine the main metabolisms driving the dissimilarity between contamination types.

## Results

RELATE analysis revealed a Spearman rank coefficient of 0.9 for the comparison between hierarchical levels 1 and 2, indicating similar results were seen irrespective of hierarchical level. Thus, to create a generalised, set of metabolic footprints, all further analyses were conducted on hierarchical level 1. 

**Figure 1:**
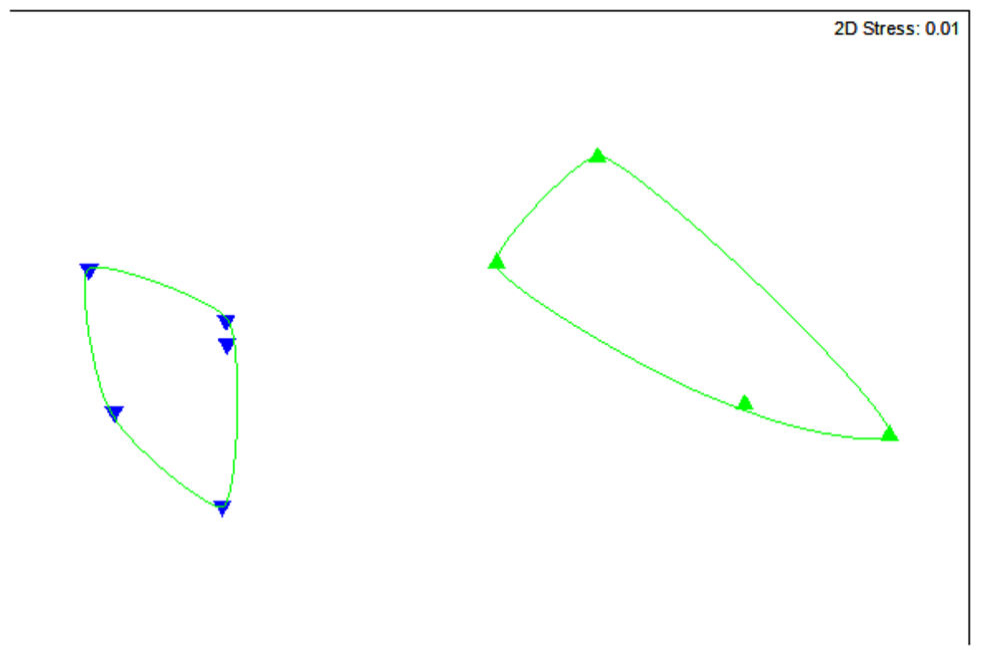
Comparison of hydrocarbon-impacted samples (green) and non-impacted samples (blue). This NMDS ordination is derived from a Bray-Curtis similarity matrix calculated from the square-root transformed abundance of DNA fragments matching the subsystems database, level hierarchical system 1 (BLASTX E-value < E<1×10^-5^). The light green polygons depict significantly different groupings (*P* < 0.05) as calculated by similarity profile (SIMPROF) analysis in PRIMER v6. See Table S1 in File S1 for the provenance of samples included in this analysis.

**Figure 2:**
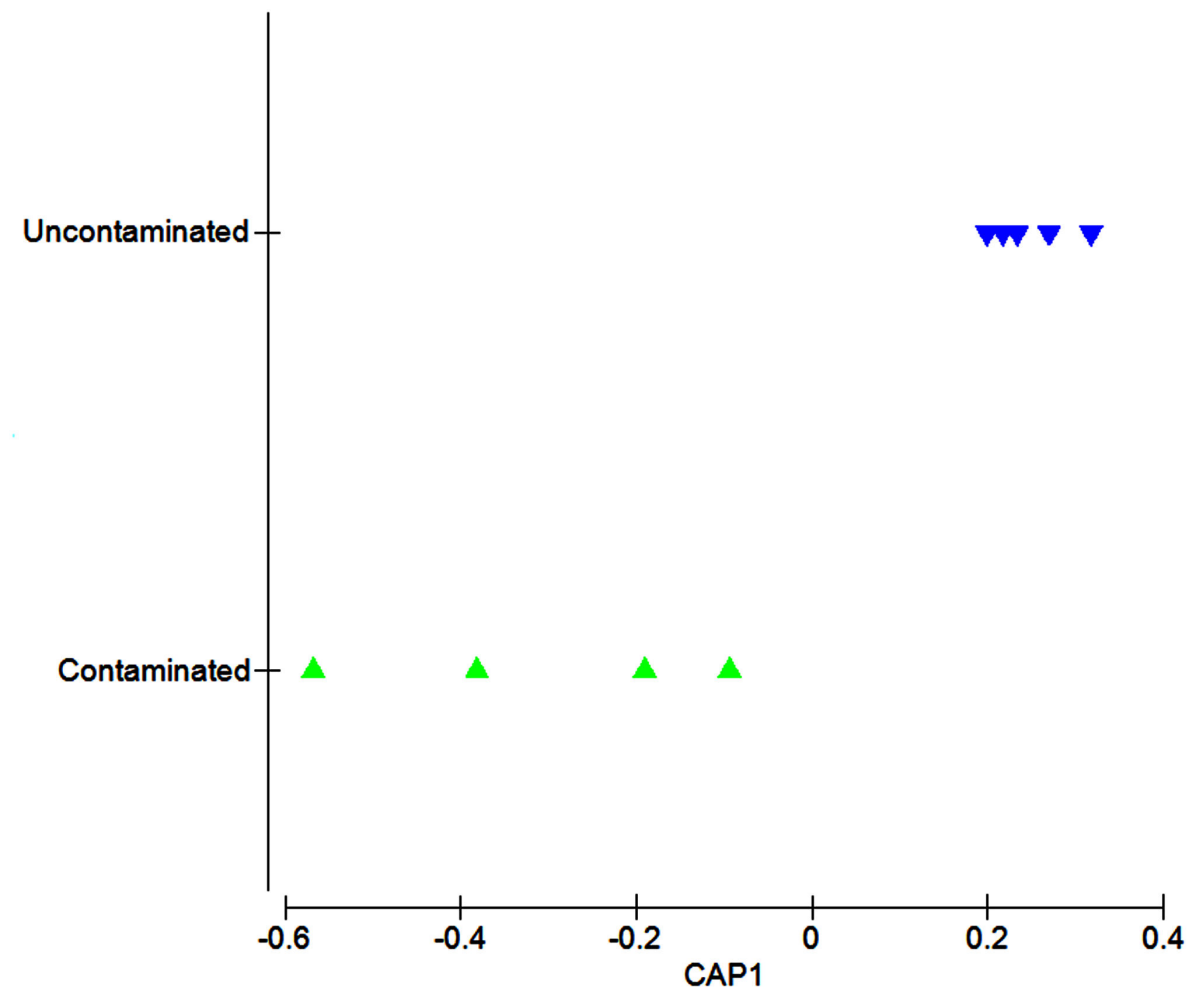
Comparison of hydrocarbon-impacted samples (green) and non-hydrocarbon-impacted samples (blue). CAP analysis (using m = 1 principal coordinate axes) is derived from the sum of squared correlations of DNA fragments matching the subsystems database, level hierarchical system 1 (BLASTX E-value < E<1×10^-5^). Significance P = 0.008 and the first axis explained δ^2^ = 0.83 of the total variation. See Table S1 in File S1 for the provenance of samples included in this analysis.

NMDS analysis revealed a clear separation of data between the hydrocarbon-impacted and non-impacted sediment metagenomes (Figure 1). CAP analysis confirmed this separation showing significant differences between the two groups (*P* = 0.008). A strong association between the multivariate data and the hypothesis of metabolic difference was indicated by the large size of their canonical correlations (δ^2^ = 0.83). The first canonical axis (m = 1) separated the sample types with no overlap (Figure 2). Cross validation of the CAP model showed all samples were correctly classified to either hydrocarbon-impacted or non-impacted sediments, hence with a zero mis-classification rate. 

SIMPER analysis revealed that the main metabolic processes contributing to the dissimilarity in the non-impacted sediments, when compared to the hydrocarbon-impacted sediments, were genes associated with cofactors, virulence, phages and fatty acids, together accounting for 45.7 % of the overall dissimilarity. Genes associated with protein metabolism, carbohydrates, amino acids, clustering-based subsystems, potassium metabolism, respiration, RNA metabolism, nucleosides and cell wall were also higher in the non-impacted site compared to the impacted sites, collectively contributing to 9.9% of the overall dissimilarity (Table 1 & S2 in File S1). 

**Table 1:** Contribution of metabolic hierarchical system level 1 to the dissimilarity of the hydrocarbon-impacted and non-hydrocarbon-impacted metagenomes.

	**Avg. Abundance**			
**Metabolic Processes**	**Hydrocarbon-Impacted**	**Non-Impacted**	**Diss/SD**	**Cum %**
Cofactors, Vitamins, Prosthetic Groups, Pigments	0.1	**0.19**	2.24	11.43
Virulence, Disease and Defence	0.1	**0.19**	2.24	22.86
Phages, Prophages, Transposable elements, Plasmids	0.1	**0.19**	2.24	34.29
Fatty Acids, Lipids, and Isoprenoids	0.1	**0.19**	2.24	45.71
Iron acquisition and metabolism	**0.84**	0.79	1.63	52.68
Dormancy and Sporulation	**0.71**	0.68	1.49	57.48
Motility and Chemotaxis	**0.83**	0.81	1.58	61.17
Metabolism of Aromatic Compounds	**0.87**	0.85	1.73	64.81
Secondary Metabolism	**0.76**	0.75	1.16	68.32
Regulation and Cell signalling	**0.86**	0.83	1.86	71.55
Protein Metabolism	0.94	**0.96**	3.42	74.53
Carbohydrates	0.97	**1**	3.5	77.49
Nitrogen Metabolism	**0.84**	0.82	1.74	80.17
Photosynthesis	0.69	0.69	1.3	82.75
Amino Acids and Derivatives	0.96	**0.98**	2.89	85.24
Clustering-based subsystems	0.98	**0.99**	1.96	87.06
Miscellaneous	0.94	**0.96**	3.14	88.7

Hydrocarbon-impacted samples include a hydrocarbon-impacted foreshore and a biopile from Australia [40; Smith et al., unpublished data], and 2 biopiles from the Arctic region [40], while the non- impacted samples included 2 marine sediment samples from Australia and 3 sediment samples from the Coorong [50]. Average dissimilarity between the two groups is 1.78 % (Table S1 in File S1). Only metabolisms that were consistent (i.e. Diss/SD > 1.4) are shown here. The larger value in each case (i.e. the potential indicator of that condition) is shown in bold.

Cut-off percentage = 90% of the total dissimilarity, Diss=dissimilarity; SD=Standard Deviation; Cum %=cumulative percentage of contribution to overall dissimilarity, Avg. Abundance values are reported for square-root transformed data

Conversely, the main metabolic processes associated with the hydrocarbon-impacted sediments were iron acquisition and metabolism, dormancy and sporulation, motility, metabolism of aromatic compounds and cell signalling accounting for 22.3 % of the overall dissimilarity between the two groups (Table 1). Genes associated with nitrogen, phosphorus and sulfur metabolism were also higher in the hydrocarbon impacted site, collectively accounting for 2.5 % of the dissimilarity to the non-impacted sites. Regardless of percent contribution, however, all metabolic processes, with the exception of secondary metabolism and photosynthesis, are likely good discriminators for hydrocarbon-impacted or non-impacted sediments, as indicated by a dissimilarity/standard deviation ratio (Diss/SD) of greater than 1.4 [46] (Tables 1 & S2 in File S1).

**Figure 3:**
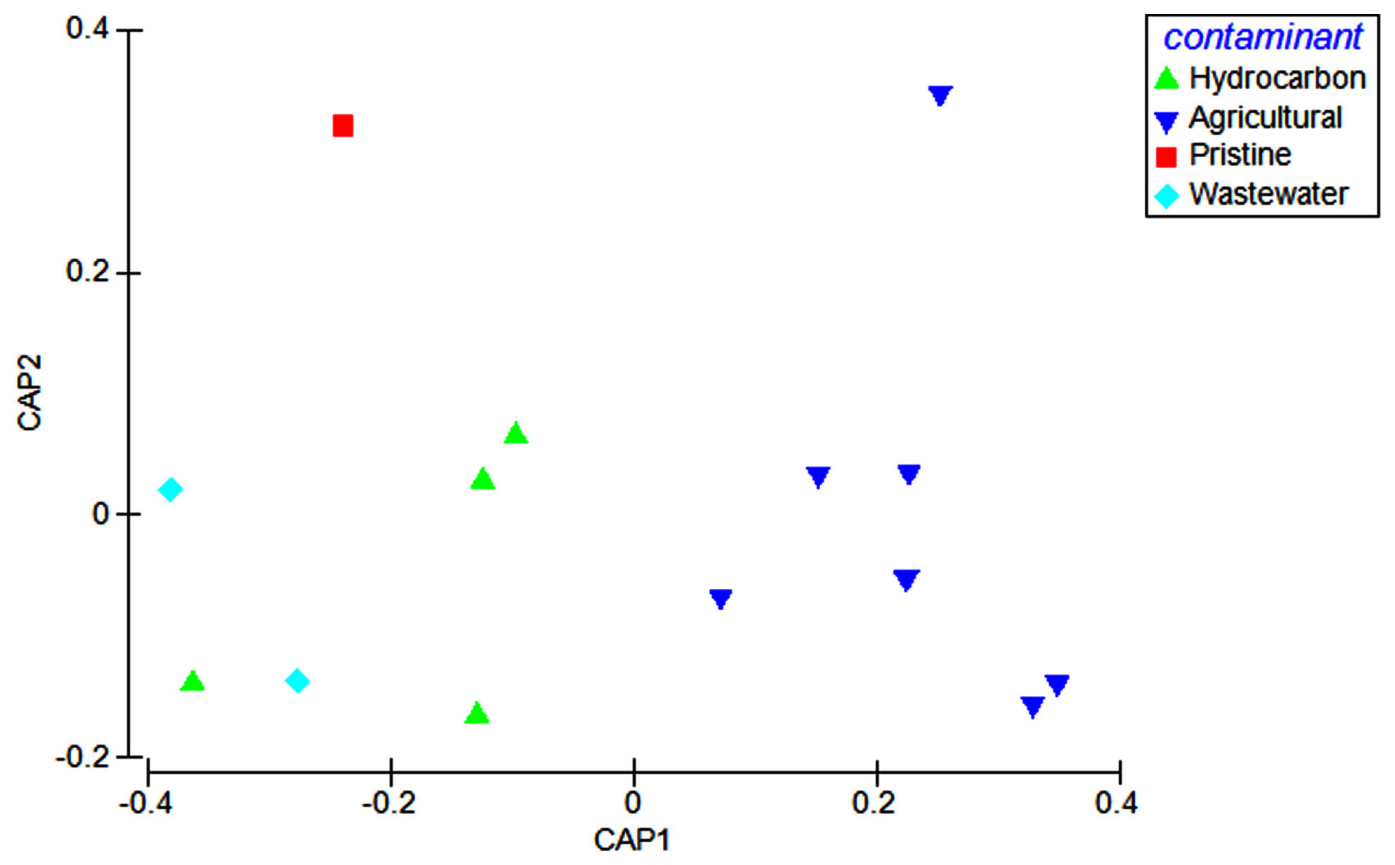
Metabolic comparison of a variety of impacted environments (**Table** **S1** in File S1). CAP analysis (using m = 2 principal coordinate axes) is derived from the sum of squared correlations of DNA fragments matching the subsystems database, level hierarchical system 1 (BLASTX E-value < E<1×10^-5^). Significance *P* = 0.0005 and the first axis explained δ^2^ = 0.88 of the total variation.

To determine if the metabolic footprints could be distinguished between contaminant types, multiple contamination types from diverse substrate types were compared (Table S1 in File S1). CAP ordination revealed a clear separation of data among the different impacted environments based on metabolic potential (Figure 3); (*P* = 0.0005) (Table 2). A strong association was seen between the multivariate data and the hypothesis of metabolic differences, indicated by the large size of their canonical correlations (hierarchial level 1: δ^2^ = 0.88). Cross validation of the CAP model showed 79% of samples overall were correctly classified to their impacted environments. More specifically, 75% and 100% of hydrocarbon and agricultural-impacted samples were correctly allocated, while only 50% and 0% of wastewater and pristine samples, respectively, were correctly classified (Table 2). 

**Table 2:** Results of CAP analysis (using *m* = 2 principal coordinate axes, explaining 88 % of total variation) testing the hypothesis that contaminant types differ for Level 1 metabolisms associated with impacted metagenomes.

**Contaminant**	**Hydrocarbon**	**Agricultural**	**Pristine**	**Wastewater**	**Total**
**Result**					
**Allocation Success %**	75	100	0	50	79
**Ratio of correct:total**	3:4	7:7	0:1	1:2	11:14
**Mis-classified to:**	Wastewater	NA	Hydrocarbon	Wastewater

Hydrocarbon-impacted samples include a hydrocarbon-impacted foreshore and a biopile from Australia [40; Smith et al., unpublished data], and 2 biopiles from the Arctic region [40], while the non- impacted samples included 2 marine sediment samples from Australia and 3 sediment samples from the Coorong [50]. Average dissimilarity between the two groups is 1.78 % (Table S1 in File S1). Only metabolisms that were consistent (i.e. Diss/SD > 1.4) are shown here. The larger value in each case (i.e. the potential indicator of that condition) is shown in bold.

Cut-off percentage = 90% of the total dissimilarity, Diss=dissimilarity; SD=Standard Deviation; Cum %=cumulative percentage of contribution to overall dissimilarity, Avg. Abundance values are reported for square-root transformed data

Significance of trace and delta statistics was *P* = 0.0005 and first canonical axis alone explained 80 % of total variation. NA = not applicable because of no mis-classifications.

Based on CAP ordinations as well as mis-classification rates, SIMPER analysis was used to determine distinguishing metabolic processes for the hydrocarbon and agricultural-impacted sites only. SIMPER analysis revealed the main metabolic processes contributing to the dissimilarity in the agriculturally-impacted environments when compared to the hydrocarbon-impacted environments were genes associated with cofactors, virulence, phages and fatty acids, collectively accounting for 48.4% of the overall dissimilarity between these two types. Genes associated with protein metabolism, carbohydrates, amino acids and clustering-based subsystems were also higher in the agricultural-impacted sites when compared to hydrocarbon-impacted sites, collectively contributing to another 9.06% of the overall dissimilarity (Tables 3 & S3 in File S1).

**Table 3:** Contribution of metabolic hierarchical system 1 to the dissimilarity of the hydrocarbon and agricultural impacted environments.

	**Avg. Abundance**			
**Metabolic Processes**	**Hydrocarbon- impacted**	**Agricultural- impacted**	**Diss/SD**	**Cum %**
Cofactors, Vitamins, Prosthetic Groups, Pigments	0.08	**0.19**	1.67	12.09
Virulence, Disease and Defence	0.08	**0.19**	1.67	24.19
Phages, Prophages, Transposable elements, Plasmids	0.08	**0.19**	1.67	36.28
Fatty Acids, Lipids, and Isoprenoids	0.08	**0.19**	1.67	48.38
Iron acquisition and metabolism	**0.84**	0.79	1.76	54.29
Dormancy and Sporulation	**0.71**	0.67	1.4	58.92
Metabolism of Aromatic Compounds	**0.87**	0.84	1.82	62.37
Motility and Chemotaxis	**0.83**	0.8	1.67	71.84
Protein Metabolism	0.93	**0.96**	3.27	74.59
Carbohydrates	0.97	**0.99**	3.44	77.27
Nitrogen Metabolism	**0.84**	0.81	1.84	82.37
Regulation and Cell signalling	**0.85**	0.83	1.81	84.78
Amino Acids and Derivatives	0.96	**0.98**	2.35	86.73
Clustering-based subsystems	0.97	**0.99**	1.75	88.4

Average dissimilarity between the two groups is 2.08 %. Only metabolisms that were consistent (i.e. Diss/SD > 1.4) are shown here. The larger value in each case (i.e. the potential indicator of that condition) is shown in bold.

Cut-off percentage = 90% of total dissimilarity, Diss=dissimilarity; SD=Standard Deviation; Cum %=cumulative percentage of contribution to overall dissimilarity, Avg. Abundance values are reported for square-root transformed data

Alternatively, the main metabolic processes associated with hydrocarbon impact were genes related to iron acquisition and metabolism, dormancy, aromatic compound degradation, and motility, collectively contributing to 17.1% of the overall dissimilarity (Table 3 & S3 in File S1). Genes associated with nitrogen metabolism and regulation were also higher in the hydrocarbon- impacted sites when compared to agricultural impacted sites, collectively accounting for 4.9% (Table 3 & S3 in File S1). Furthermore, all metabolic processes, with the exception of photosynthesis, secondary metabolism and potassium metabolism were consistently distinguishable between agricultural and hydrocarbon-impacted environments, as indicated by a Diss/SD of greater than 1.4 [46].

## Discussion

Microbial communities are known to respond to hydrocarbon contamination at the functional level, whereby a shift in metabolic potential can be observed [14,47,48]. Thus, a major goal in the study of bioremediation is to identify the key metabolic processes being undertaken by the inhabiting microbial communities [38,49]. Here, we report the first metagenomic study to identify the overall metabolic footprints associated with discriminating hydrocarbon-impacted versus non-impacted sediment samples. 

### The metabolic footprints of hydrocarbon degradation

RELATE analysis showed a significant correlation (Rho: 0.773; *P* < 0.002) between hierarchial level 1 and 2, indicating there is no significant loss of information between the different levels of resolution. This result is consistent with previous studies that have shown changes to environmental conditions caused by anthropogenic disturbances have led to major shifts in microbial community functionality that become evident across multiple levels of resolution [6,8,50]. 

Unconstrained (NMDS) and constrained (CAP) multivariate analyses, both showed clear separation of data (*P*-value = 0.008) between the hydrocarbon-impacted and non-impacted sediments (Figures 1 & 2). The similarities between constrained and unconstrained ordinations likely reflect the hydrocarbon impact. This is supported by the CAP analysis, which shows that the majority of the variance is expressed on just the first canonical axis, with a squared canonical correlation (δ^2^) of 0.83. A recent hydrocarbon-based study used high throughput functional gene array technology to show that all microbial samples with hydrocarbon contamination grouped together indicative of similar functional patterns [31]. Furthermore, it has been shown that differences in metabolic processes could be used to predict the biogeochemical status of the environment [4]. Thus, the clear separation between data points in the NMDS and CAP plots indicates the hydrocarbon-impacted sediment samples can be readily distinguished even at this coarse level of metabolic resolution, despite differences in geographical location. Furthermore, the same separation seen in unconstrained and constrained ordination methods demonstrates that the data points are not simply conforming to the more hypothesis-driven CAP analysis.

The majority of the separation between the hydrocarbon-impacted and non-impacted groups was explained by a higher relative abundance of genes associated with cofactors, virulence, phages and fatty acids, collectively accounting for nearly half of the dissimilarity in the non-impacted sediment samples when compared to the impacted sites (Table 1). Those microbes capable of surviving following hydrocarbon impact become dominant, eventually leading to a major shift in the structure of the community [32,51]. This shift in structure is generally coupled with a shift in functionality, whereby previous studies have shown a significant decrease in the overall microbial functional diversity [6,31,52]. Thus, the high degree of dissimilarity driven by the non-impacted sediments, suggests the major factor causing the differences between the two groups can be explained by a shift in functionality, which has led to the reduction in non-essential metabolisms following hydrocarbon impact. 

The reduction in non-essential metabolic pathways was coupled with a subsequent increase in pathways associated iron acquisition and metabolism, dormancy and sporulation, motility, metabolism of aromatic compounds and cell signalling (Table 1). These pathways have all previously been linked to stressed environments [6,53–55], suggesting the microbial communities inhabiting the hydrocarbon-impacted environments are expending more energy on pathways essential to the utilization of carbon and survival. 

The degradation of hydrocarbons is often hindered by the requirement to come into direct contact with hydrocarbon substrates [56]. Therefore, many microorganisms capable of catabolising hydrocarbons have shown chemotaxis abilities allowing them to move towards, and subsequently degrade the contaminant at a higher rate [57–59]. This degradation ability is then often further enhanced by the secretion of biosurfactants, which increase the availability of hydrocarbons in the soil [60]. Thus, the increase in motility and chemotaxis genes suggest that the microbial communities are increasing metabolic pathways that will allow for direct contact with hydrocarbon compounds (Table 1).

Following direct contact, the microbial communities must have genes that allow for the catabolism of hydrocarbons. Petroleum hydrocarbons are comprised of a complex mixture of compounds including cycloalkanes, alkanes, polycyclic aromatic hydrocarbons, aromatics and phenolics [61]. Previous studies have shown an increase in genes associated with the breakdown of these compounds in hydrocarbon-contaminated environments [31,62]. Thus, a higher relative abundance of metabolism of aromatic compound genes in the hydrocarbon-impacted sediments when compared to the non-impacted sediments is consistent with a community optimising its ability to utilise hydrocarbon as an energy source (Table 1). 

Following hydrocarbon contamination, microbial communities must adapt to survive the sudden increase in carbon availability and subsequent loss of limiting nutrients such as nitrogen and phosphorus and in some cases iron [14,55,63]. As a result, an increase in genes associated with nitrogen, phosphorus and iron metabolism have been shown, allowing for effective scavenging mechanisms (Smith et al., unpublished data). Our results indicate there may have been an increased need for nitrogen, phosphorus and iron metabolites in the hydrocarbon-impacted sediments when compared to non-impacted sediments. Furthermore, genes associated with cofactors, amino acid pathways, carbohydrates and protein metabolisms were all reduced in the hydrocarbon-impacted sites (Tables 1 & S2 in File S1). Taken together, these results suggest that the microbial communities are expending most of their energy scavenging key nutrients needed for bioremediation of hydrocarbons, leading to the subsequent decrease in pathways associated with more complex carbohydrate and protein metabolisms and growth. 

### Contaminant types

When the hydrocarbon-impacted environments were compared to metagenomes experiencing different contaminant types from a wide range of habitats, CAP analysis showed a significant difference (*P*-value = 0.0005; Table 2) between the relative abundances of metabolisms across these impacted environments (Figure 3). In particular, hydrocarbon and agricultural-impacted environments were found to have the highest allocation success, 75% and 100% respectively, when compared to wastewater and pristine sites, 50% and 0%, respectively (Table 2). The higher allocation success for hydrocarbon and agricultural impacted sites was likely driven by the larger sample size for these environments. Furthermore, as the metagenomic samples included were from a variety of substrate types and geographical locations (Table S1 in File S1), our results indicate that the metabolic footprints created due to a contamination event, were more significant when compared to differences created based on geographical location and physico-chemical conditions (Table S4 in File S1).

SIMPER analysis revealed the main distinguishing metabolic processes associated with agricultural impacted environments were genes associated with cofactors, virulence, phages, fatty acids, protein metabolism, carbohydrates, amino acids and clustering based subsystems (Tables 3 & S3 in File S1), collectively accounting for 57.4% of the overall dissimilarity from the hydrocarbon-impacted environments. Agricultural practices are known to increase the deposition of nutrients into the surrounding environment [64,65]. Previous studies have shown that an increase of nutrients via agricultural impact can lead to an increase in microbial productivity [8]. As previously discussed, hydrocarbon impact has been shown to lead to a reduction in genotypic diversity, whereby only the essential metabolisms remain [6,31]. This is thought to be due to the toxic effect of hydrocarbon pollution which in turn can lead to a community exerting more energy for survival than on growth and productivity [66]. Thus, an increase in genes associated with protein metabolism in the agricultural impacted environments (Table 3) is consistent with a more active community when compared to the hydrocarbon-impacted environments [67]. 

SIMPER analysis also revealed the main distinguishing metabolic processes associated with hydrocarbon-impacted environments was a higher relative abundance of genes associated with iron acquisition and metabolism, dormancy, aromatic compound degradation, motility, nitrogen metabolism and regulation, collectively contributing to 22.1% of the overall dissimilarity (Table 3). These results are consistent with SIMPER results when comparing hydrocarbon-impacted and non-impacted environments, indicating the metabolic footprints for contaminant types are consistent even at this coarse level of metabolic resolution. Furthermore, hydrocarbon-impacted and agricultural-impacted metabolic footprints were distinguishable irrespective of differences in substrate type, physico-chemical conditions and geographical location. Thus, CAP analysis suggests these impacted environments have acquired microbial communities with differing metabolic functions, which have allowed for our ability to distinguish between contaminant types.

Although some pathways contributed to the dissimilarity between the two groups more than others, all metabolisms with the exception of photosynthesis and potassium metabolism (at the 90% cut off percentage) were identified as being consistent distinguishing metabolisms (Tables 1, 3, S2 & S3 in File S1). This suggests all are metabolic footprints of their given environment, indicating the overall metabolic signature is different between groups. In nature, microbial communities are typically composed of mixed communities characterised by an intricate network of metabolic processes [23]. Consequently, our results indicate a complete overview of the metabolites present within the inhabiting microbial consortia is needed to effectively characterise an environment. 

## Conclusion

Our approach indicates the hydrocarbon-impacted sediment samples can be distinguished from non-impacted sediments based on their metabolic signatures despite differences in geographical location. These signatures include metabolisms associated with iron acquisition and metabolism, dormancy and sporulation, motility, metabolism of aromatic compounds, cell signalling and nitrogen, phosphorus and sulfur metabolism. Our analysis also indicated that the majority of the dissimilarity was, however, due to a reduction of functional genes associated with cofactors, virulence, phages and fatty acids. Further to this, our approach illustrates the ability to distinguish between contaminant types from a wide range of habitats, with a clear separation in data points associated with either hydrocarbon or agricultural contamination. Here we provide the first metagenomic study to elucidate the metabolic footprints associated with hydrocarbon impact. Furthermore, the differentiation between hydrocarbon contaminants, for example long chain hydrocarbons compared to aromatics, is needed to fully determine the effects of hydrocarbon impacts on the environment. 

## Supporting Information

File S1 Supporting Information.
(DOCX)

## References

[1] Falkowski PG, Fenchel T, Delong EF (2008) The Microbial Engines That Drive Earth’s Biogeochemical. Cycles - Microbial Ecology 320: 1034 - 1039. 10.1126/science.1153213 18497287

[2] Chapin FS III, Walker BH, Hobbs RJ, Hooper DU, Lawton JH et al. (1997) Biotic control over the functioning of ecosystems. Science 277: 500 - 504. doi:10.1126/science.277.5325.500.

[3] Karl DM (2002) Nutrient dynamics in the deep blue sea. Trends Microbiol 10: 410 - 418. doi:10.1016/S0966-842X(02)02430-7. PubMed: 12217506. 12217506

[4] Dinsdale EA, Edwards RA, Hall D, Angly F, Breitbart M et al. (2008) Functional metagenomic profiling of nine biomes. Nature 452: 629 - 633. doi:10.1038/nature06810. PubMed: 18337718. 18337718

[5] Brown JH, Gillooly JF, Allen AP, Savage VM (1771 - 1789) West GB (2004) Toward a metabolic theory of ecology. Ecology 85.

[6] Hemme CL, Deng Y, Gentry TJ, Fields MW, Wu L et al. (2010) Metagenomic insights into evolution of a heavy metal-contaminated groundwater microbial community.ISME J 4: 660-672. doi:10.1038/ismej.2009.154. PubMed: 20182523. 20182523

[7] Kostka JE, Prakash O, Overholt WA, Green SJ, Freyer G et al. (2011) Hydrocarbon-Degrading Bacteria and the Bacterial Community Response in Gulf of Mexico Beach Sands Impacted by the Deepwater Horizon Oil Spill. Appl Environ Microbiol 77: 7962 - 7974. doi:10.1128/AEM.05402-11. PubMed: 21948834. 21948834 PMC3208977

[8] Smith RJ, Jeffries TC, Roudnew B, Fitch AJ, Seymour JR et al. (2012) Metagenomic comparison of microbial communities inhabiting confined and unconfined aquifer ecosystems. Environ Microbiol 14: 240 - 253. PubMed: 22004107. 22004107 10.1111/j.1462-2920.2011.02614.x

[9] Henriques IDS, Aga DS, Mendes P, Connor SKO, Love NG (2007) Metabolic Footprinting: A New Approach to Identify Physiological Changes in Complex Microbial Communities upon Exposure to Toxic Chemicals. Environ Sci Technol 41: 3945 - 3951. doi:10.1021/es062796t. PubMed: 17612173. 17612173

[10] Gianoulis TA, Raes J, Patel PV, Bjornson R, Korbel JO et al. (2009) Quantifying environmental adaptation of metabolic pathways in metagenomics. Proc Natl Acad Sci U S A 106: 1374 - 1379. doi:10.1073/pnas.0808022106. PubMed: 19164758. 19164758 PMC2629784

[11] Röling WFM, Ferrer M, Golyshin PN (2010) Systems approaches to microbial communities and their functioning. Curr Opin Biotechnol 21: 532 - 538. doi:10.1016/j.copbio.2010.06.007. PubMed: 20637597. 20637597

[12] Schimel J, Balser TC, Wallenstein M (2007) Microbial stress-response physiology and its implications for ecosystem function. Ecology 88: 1386 - 1394. doi:10.1890/06-0219. PubMed: 17601131. 17601131

[13] Steube C, Richter S, Griebler C (2009) First attempts towards an integrative concept for the ecological assessment of groundwater ecosystems. Hydrogeol J 17: 23 - 35. doi:10.1007/s10040-008-0346-6.

[14] Head IM, Jones DM, Röling WFM (2006) Marine microorganisms make a meal of oil. Nat Rev Microbiol 4: 173 - 182. doi:10.1038/nrmicro1348. PubMed: 16489346. 16489346

[15] Mailaa MP, Cloeteb TE (2005) The use of biological activities to monitor the removal of fuel contaminants—perspective for monitoring hydrocarbon contamination: a review. Int Biodeterior 55: 1 - 8. doi:10.1016/j.ibiod.2004.10.003.

[16] Avidano L, Gamalero E, Cossa GP, Carraro E (2005) Characterization of soil health in an Italian polluted site by using microorganisms as bioindicators. Appl Soil Ecol 30: 21-33. doi:10.1016/j.apsoil.2005.01.003.

[17] Anderson T (2003) Microbial eco-physiological indicators to asses soil quality. Agric Ecosyst Environ 98: 285 - 293. doi:10.1016/S0167-8809(03)00088-4.

[18] Bonjoch X, Ballesté E, Blanch AR (2004) Multiplex PCR with 16S rRNA Gene-Targeted Primers of *Bifidobacterium* spp. To Identify Sources of Fecal Pollution. Appl Environ Microbiol 70: 3171 - 3175. doi:10.1128/AEM.70.5.3171-3175.2004. PubMed: 15128586. 15128586 PMC404414

[19] Wooley JC, Ye Y (2010) Metagenomics: Facts and Artifacts, and Computational Challenges. J Comput Sci Technol 25: 71 - 81. doi:10.1007/s11390-010-9306-4. PMC2905821 20648230

[20] Buyer JS, Drinkwater LE (1997) Comparison of substrate utilization assay and fatty acid analysis of soil microbial communities. J Microbiol Methods 30: 3 - 11. doi:10.1016/S0167-7012(97)00038-9.

[21] Hernesmaa A, Björklöf K, Kiikkilä O, Fritze H, Haahtela K et al. (2005) Structure and function of microbial communities in the rhizosphere of Scots pine after tree-felling. Soil Biol Biochem 37: 777 - 785. doi:10.1016/j.soilbio.2004.10.010.

[22] Williams BK, Titus K (1988) Assessment of sampling stability in ecological applications of discriminant analysis. Ecology 69: 1275 - 1285. doi:10.2307/1941283.

[23] Pelz O, Tesar M, Wittich RM, Moore ERB, Timmis KN et al. (1999) Towards elucidation of microbial community metabolic pathways: unravelling the network of carbon sharing in a pollutant-degrading bacterial consortium by immunocapture and isotopic ratio mass spectrometry. Environ Microbiol 1: 167 - 174. doi:10.1046/j.1462-2920.1999.00023.x. PubMed: 11207732. 11207732

[24] Anderson MJ, Willis TJ (2003) Canonical analysis of principle coordinates: A useful method of constrained ordination for ecology. Ecology 84: 511 - 525. Available online at: doi:10.1890/0012-9658(2003)084[0511:CAOPCA]2.0.CO;2

[25] Bastias BA, Huang ZQ, Blumfield T, Xu Z, Cairney JWG (2006) Influence of repeated prescribed burning on the soil fungal community in an eastern Australian wet sclerophyll forest. Soil Biol Biochem 38: 3492 - 3501. doi:10.1016/j.soilbio.2006.06.007.

[26] Cookson WR, Osman M, Marschner P, Abaye DA, Clark I et al. (2007) Controls on soil nitrogen cycling and microbial community composition across land use and incubation temperature. Soil Biol Biochem 39: 744 - 756. doi:10.1016/j.soilbio.2006.09.022.

[27] Lear G, Lewis GD (2009) Impact of catchment land use on bacterial communities within stream biofilms. Ecol Indic 9: 848 - 855. doi:10.1016/j.ecolind.2008.10.001.

[28] Baker KL, Langenheder S, Nicol GW, Ricketts D, Killham K et al. (2009) Environmental and spatial characterisation of bacterial community composition in soil to inform sampling strategies. Soil Biol Biochem 41: 2292 - 2298. doi:10.1016/j.soilbio.2009.08.010.

[29] Singleton I (1994) Microbial Metabolism of Xenobiotics: Fundamental and Applied Research. J Chem Technol Biotechnol 59: 9 - 23. doi:10.1002/jctb.280590104.

[30] Chikere CB, Okpokwasili GC, Chikere BO (2011) Monitoring of microbial hydrocarbon remediation in the soil. 3 Biotech 1: 117 - 138 10.1007/s13205-011-0014-8 PMC3339601 22611524

[31] Liang Y, Van Nostrand JD, Deng Y, He Z, Wu L et al. (2011) Functional gene diversity of soil microbial communities from five oil-contaminated fields in China.ISME J 5: 403 - 413. doi:10.1038/ismej.2010.142. PubMed: 20861922. 20861922 PMC3105718

[32] Viñas M, Sabaté J, José Espuny M, Solanas AM (2005) Bacterial Community Dynamics and Polycyclic Aromatic Hydrocarbon Degradation during Bioremediation of Heavily Creosote-Contaminated Soil. Appl Environ Microbiol 71: 7008 - 7018. doi:10.1128/AEM.71.11.7008-7018.2005. PubMed: 16269736. 16269736 PMC1287751

[33] Röling WFM, Milner MG, Martin Jones D, Lee K, Daniel F et al. (2002) Robust Hydrocarbon Degradation and Dynamics of Bacterial Communities during Nutrient-Enhanced Oil Spill Bioremediation. Appl Environ Microbiol 68: 5537 - 5548. doi:10.1128/AEM.68.11.5537-5548.2002. PubMed: 12406747. 12406747 PMC129918

[34] Singh AK, Sherry A, Gray ND, Jones MD, Röling WFM, et al. (2011) Dynamics of *Alcanivorax* spp. in Oil-Contaminated Intertidal Beach Sediments Undergoing Bioremediation. Appl Microbiol Mol Biol DOI 10.1007/978-90-481-9252-6_24: 199 - 209

[35] Xu R, Obbard JP, Tay ETC (2003) Optimization of slow-release fertilizer dosage for bioremediation of oil-contaminated beach sediment in a tropical environment. World J Microbiol Biotechnol 19: 719 - 725. doi:10.1023/A:1025116421986.

[36] Yakimov MM, Timmis KN, Golyshin PN (2007) Obligate oil-degrading marine bacteria. Curr Opin Biotechnol 18: 257 - 266. doi:10.1016/j.copbio.2007.04.006. PubMed: 17493798. 17493798

[37] Walworth J, Pond A, Snape I, Rayner J, Ferguson S et al. (2007) Nitrogen requirements for maximizing petroleum bioremediation in a sub-Antarctic soil. Cold Reg Sci Technol 48: 84 - 91. doi:10.1016/j.coldregions.2006.07.001.

[38] Chakraborty R, Wu CH, Hazen TC (2012) Systems biology approach to bioremediation. Curr Opin Biotechnol 23: 1 - 8. doi:10.1016/j.copbio.2011.12.020. PubMed: 22244690. 22342400

[39] Kennedy J, Flemer B, Jackson SA, Lejon DPH, Morrissey JP et al. (2010) Marine Metagenomics: New Tools for the Study and Exploitation of Marine Microbial. Metabolism - Mar Drugs 8: 608 - 628. doi:10.3390/md8030608. 20411118 PMC2857354

[40] Yergeau E, Sanschagrin S, Beaumier D, Greer CW (2012) Metagenomic Analysis of the Bioremediation of Diesel-Contaminated Canadian High Arctic Soils. PLOS ONE 7: 1 - 10. PubMed: 22253877. 10.1371/journal.pone.0030058 PMC3256217 22253877

[41] Meyer F, Paarmann D, D'Souza M, Olsem R, Glass EM et al. (2008) The metagenomics RAST server – a public resource for the automatic phylogenetic and functional analysis of metagenomes. BMC Bioinformatics 9: 1 - 8. doi:10.1186/1471-2105-9-S10-O1. PubMed: 18173834. 18803844 PMC2563014

[42] Clarke A, Gorley R (2006) PRIMER v6: User Manula/Tutorial. Plymouth, UK: PRIMER-E..

[43] Clarke KR (1993) Nonparametric multivariate analysis of changes in community structure. Aust J Ecol 18: 117 - 143. doi:10.1111/j.1442-9993.1993.tb00438.x.

[44] Anderson MJ, Robinson J (2001) Permutation tests for linear models. Aust NZ J Stat 43: 75 - 88. doi:10.1111/1467-842X.00156.

[45] Anderson MJ, Gorley RN, Clarke KR (2008) PERMANOVA+ for PRIMER: Guide to Software and Statistical Methods. PRIMER-E, Plymouth, UK.

[46] Clarke KR, Warwick RM (2001) Change in Marine Communities: An Approach to Statistical Analysis and Interpretation, 2nd Edition. PRIMER-E Ltd.

[47] Langworthy DE, Stapleton RD, Sayler GS, Findlay RH (1998) Genotypic and Phenotypic Responses of a Riverine Microbial Community to Polycyclic Aromatic Hydrocarbon Contamination. Appl Environ Microbiol 64: 3422 - 3428. PubMed: 9726892 10.1128/aem.64.9.3422-3428.1998 PMC106742 9726892

[48] Siciliano SD, Germida JJ, Banks K, Greer CW (2003) Changes in Microbial Community Composition and Function during a Polyaromatic Hydrocarbon Phytoremediation Field. Trial - Appl Environ Microbiol 69: 483 - 489. 12514031 10.1128/AEM.69.1.483-489.2003 PMC152433

[49] Watanabe K (2001) Microorganisms relevant to bioremediation. Curr Opin Biotechnol 12: 237 - 241. doi:10.1016/S0958-1669(00)00205-6. PubMed: 11404100. 11404100

[50] Jeffries TC, Seymour JR, Gilbert JA, Dinsdale EA, Newton K, et al. (2011) Substrate Type Determines Metagenomic Profiles from Diverse Chemical Habitats. PLoS ONE 6: e25173 25110.21371/journal.pone.0025173 21966446 10.1371/journal.pone.0025173 PMC3179486

[51] Wu Y, Luo Y, Zou D, Ni J, Liu W et al. (2008) Bioremediation of polycyclic aromatic hydrocarbons contaminated soil with Monilinia sp.: degradation and microbial community analysis. Biodegradation 19: 247 - 257. doi:10.1007/s10532-007-9131-9. PubMed: 17541708. 17541708

[52] Liang Y, Nostrand JD, Wang J, Zhang X, Zhou J et al. (2009) Microarray-based functional gene analysis of soil microbial communities during ozonation and biodegradation of crude oil. Chemosphere 75: 193 - 199. doi:10.1016/j.chemosphere.2008.12.007. PubMed: 19144375. 19144375

[53] Ford TE (2000) Response of marine microbial communities to anthropogenic stress. J Aquat Ecosyst Stress and Recovery 7: 75 - 89. doi:10.1023/A:1009971414055.

[54] Suenaga H, Ohnuki T, Miyazaki K (2007) Functional screening of a metagenomic library for genes involved in microbial degradation of aromatic compounds. Environ Microbiol 9: 2289 - 2297. doi:10.1111/j.1462-2920.2007.01342.x. PubMed: 17686025. 17686025

[55] Schneiker S, Martins dos Santos VAP, Bartels D, Bekel T, Brecht M et al. (2006) Genome sequence of the ubiquitous hydrocarbon-degrading marine bacterium Alcanivorax borkumensis. Nat Biotechnol 24: 997 - 1004. doi:10.1038/nbt1232. PubMed: 16878126. 16878126 PMC7416663

[56] Ron EZ, Rosenberg E (2002) Biosurfactants and oil bioremediation. Curr Opin Biotechnol 13: 249 - 252. doi:10.1016/S0958-1669(02)00316-6. PubMed: 12180101. 12180101

[57] Peng RH, Xiong AS, Xue Y, Fu XY, Gao F et al. (2008) Microbial biodegradation of polyaromatic hydrocarbons. FEMS Microbiol Rev 32: 927 - 955. doi:10.1111/j.1574-6976.2008.00127.x. PubMed: 18662317. 18662317

[58] Ortega-Calvo JJ, Marchenko AI, Vorobyov AV, Borovick RV (2003) Chemotaxis in polycyclic aromatic hydrocarbon-degrading bacteria isolated from coal-tar- and oil-polluted rhizospheres. FEMS Microbiol Ecol 44: 373 - 381. doi:10.1016/S0168-6496(03)00092-8. PubMed: 19719618. 19719618

[59] Fernández-Luqueño F, Valenzuela-Encinas C, Marsch R, Martínez-Suárez C, Vázquez-Núñez E et al. (2011) Microbial communities to mitigate contamination of PAHs in soil—possibilities and challenges: a review. Environ Sci Poll R 18: 12 - 30. doi:10.1007/s11356-010-0371-6. 20623198

[60] Venkata Mohan S, Kisa T, Ohkuma T, Kanaly RA, Shimizu Y (2006) Bioremediation technologies for treatment of PAH-contaminated soil and strategies to enhance process efficiency. Rev Environ Sci Biotechnol 5: 347 - 374. doi:10.1007/s11157-006-0004-1.

[61] Hamamura N, Olson SH, Ward DM, Inskeep WP (2006) Microbial Population Dynamics Associated with Crude-Oil Biodegradation in Diverse Soils. Appl Environ Microbiol 72: 6316 - 6324. doi:10.1128/AEM.01015-06. PubMed: 16957258. 16957258 PMC1563594

[62] Yergeau E, Arbour M, Brousseau R, Juck D, Lawrence JR et al. (2009) Microarray and Real-Time PCR Analyses of the Responses of High-Arctic Soil Bacteria to Hydrocarbon Pollution and Bioremediation Treatments. Appl Environ Microbiol 75: 6258 - 6267. doi:10.1128/AEM.01029-09. PubMed: 19684169. 19684169 PMC2753079

[63] Beller HR, Grbić-Galić D, Reinhard M (1992) Microbial Degradation of Toluene under Sulfate-Reducing Conditions and the Influence of Iron on the Process. Appl Environ Microbiol 58: 786 - 793. PubMed: 1575481. 1575481 10.1128/aem.58.3.786-793.1992 PMC195335

[64] Haberl H, Erb KH, Krausmann F, Gaube V, Bondeau A, et al. (2007) Quantifying and mapping the human appropriation of net primary production in earth’s terrestrial ecosystems. Proc Natl Acad Sci USA 104: 12942 - 12947 10.1073/pnas.0704243104 PMC1911196 17616580

[65] Barnosky AD, Hadly EA, Bascompte J, Berlow EL, Brown JH et al. (2012) Approaching a state shift in Earth’s biosphere. Nature 486: 52 - 58. doi:10.1038/nature11018. PubMed: 22678279. 22678279

[66] Delille D, Delille B (2000) Field observations on the variability of crude oil impact on indigenous hydrocarbon-degrading bacteria from sub-Antarctic intertidal sediments. Marine. Environ Rev 49: 403 - 417. 10.1016/s0141-1136(99)00080-x 11285720

[67] Urich T, Lanzén A, Qi J, Huson DH, Schleper C et al. (2008) Simultaneous Assessment of Soil Microbial Community Structure and Function through Analysis of the meta-Transcriptome. PLOS ONE 3: 1 - 13. PubMed: 18575584. 10.1371/journal.pone.0002527 PMC2424134 18575584

