# Supplementary material for: Determining the Metabolic Footprints of Hydrocarbon Degradation Using Multivariate Analysis
Source: PLoS One. 2013 Nov 25;8(11):e81910. doi: 10.1371/journal.pone.0081910 (PMC3839897; doi:10.1371/journal.pone.0081910)
Supplement: File S1 — Supporting Information. (DOCX) [file pone.0081910.s001.docx]

**Supporting Information**

**Table S1 – Summary of publicly available metagenomes used in this study.**

| **MG-RAST ID** | **Description / Reference** | **Habitat Type** | **Contaminant** | **Hemisphere** | **Used in first analysis** | **Used in second analysis** |
| --- | --- | --- | --- | --- | --- | --- |
| **4453082.3** | Hydrocarbon contaminated foreshore  / Smith et al., unpublished data | Beach sediment | Hydrocarbon | Southern | X | X |
| **4453072.3** | Hydrocarbon contaminated biopile  [68] | Soil | Hydrocarbon | Southern | X | X |
| **4449126.3** | Biopiles 2006 [40] | Soil | Hydrocarbon | Northern | X | X |
| **4450729.3** | Biopile 2005 [40] | Soil | Hydrocarbon | Northern | X | X |
| **4446341.3** | Marine Sediment 1[50] | Beach sediment | Agricultural | Southern | X | X |
| **4446341.3** | Marine Sediment 2 [50] | Beach sediment | Agricultural | Southern | X | X |
| **4440984.3** | Coorong sediment 1 [50] | Sediment | Agricultural | Southern | X | X |
| **4441020.3** | Coorong sediment 2 [50] | Sediment | Agricultural | Southern | X | X |
| **4441021.3** | Coorong sediment 3[50] | Sediment | Agricultural | Southern | X | X |
| **4441022.3** | Coorong sediment 4[50] | Sediment | Agricultural | Southern |  | X |
| **4453064.3** | Unconfined aquifer [8] | Groundwater | Agricultural | Southern |  | X |
| **4453083.3** | Confined aquifer [8] | Groundwater | Pristine | Southern |  | X |
| **4455295.3** | Wastewater 1 [69] | Wastewater | Wastewater | Northern |  | X |
| **4463936.3** | Wastewater 2 [69] | Wastewater | Wastewater | Northern |  | X |

**Table S2 - Contribution of metabolic hierarchical system level 1 to the dissimilarity of the hydrocarbon-impacted and non-impacted sediment metagenomes.** Shows all metabolisms, including inconsistent ones (i.e. Diss/SD < 1.4). Average dissimilarity between the two groups is 1.78%. Bold values show either the condition with the higher average abundance (i.e. a potential indicator of that condition) or Diss/SD ratios that are consistent (i.e. > 1.4).

|  | **Avg. Abundance** | |  |  |
| --- | --- | --- | --- | --- |
| **Metabolic Processes** | **Hydrocarbon-Impacted** | **Non-Impacted** | **Diss/SD** | **Cum %** |
| Cofactors, Vitamins, Prosthetic Groups, Pigments | 0.1 | **0.19** | **2.24** | 11.43 |
| Virulence, Disease and Defence | 0.1 | **0.19** | **2.24** | 22.86 |
| Phages, Prophages, Transposable elements, Plasmids | 0.1 | **0.19** | **2.24** | 34.29 |
| Fatty Acids, Lipids and Isoprenoids | 0.1 | **0.19** | **2.24** | 45.71 |
| Iron acquisition and metabolism | **0.84** | 0.79 | **1.63** | 52.68 |
| Dormancy and Sporulation | **0.71** | 0.68 | **1.49** | 57.48 |
| Motility and Chemotaxis | **0.83** | 0.81 | **1.58** | 61.17 |
| Metabolism of Aromatic Compounds | **0.87** | 0.85 | **1.73** | 64.81 |
| Secondary Metabolism | 0.76 | 0.75 | 1.16 | 68.32 |
| Regulation and Cell signalling | **0.86** | 0.83 | **1.86** | 71.55 |
| Protein Metabolism | 0.94 | **0.96** | **3.42** | 74.53 |
| Carbohydrates | 0.97 | **1** | **3.5** | 77.49 |
| Nitrogen Metabolism | **0.84** | 0.82 | **1.74** | 80.17 |
| Photosynthesis | 0.69 | 0.69 | 1.3 | 82.75 |
| Amino Acids and Derivatives | 0.96 | **0.98** | **2.89** | 85.24 |
| Clustering-based subsystems | 0.98 | **0.99** | **1.96** | 87.06 |
| Miscellaneous | 0.94 | **0.96** | **3.14** | 88.7 |
| Potassium metabolism | 0.79 | **0.8** | **1.45** | 90.27 |
| Respiration | 0.89 | **0.9** | **1.51** | 91.79 |
| Phosphorus Metabolism | **0.84** | 0.83 | **1.41** | 93.3 |
| RNA Metabolism | 0.92 | **0.93** | **1.83** | 94.62 |
| Sulfur Metabolism | **0.84** | 0.83 | **1.6** | 95.89 |
| Nucleosides and Nucleotides | 0.88 | **0.89** | **1.58** | 97.03 |
| Cell Wall and Capsule | 0.91 | **0.92** | **1.62** | 97.74 |
| Stress Response | 0.89 | 0.89 | **1.43** | 98.38 |
| Cell Division and Cell Cycle | 0.84 | 0.84 | 1.39 | 98.99 |
| DNA Metabolism | 0.91 | 0.91 | 1.24 | 99.54 |
| Membrane Transport | 0.9 | 0.9 | 1.28 | 100 |

Diss=dissimilarity; SD=Standard Deviation; Cum %=cumulative percentage of contribution to overall dissimilarity, Avg. Abundance values are reported for square-root transformed data

**Table S3 – Contribution of metabolic hierarchical system level 1 to the dissimilarity of the hydrocarbon and agricultural impacted environments.** Shows all metabolisms, including inconsistent ones (i.e. Diss/SD < 1.4). Average dissimilarity between the two groups is 2.08%. Bold values show either the condition with the higher average abundance (i.e. a potential indicator of that condition) or Diss/SD ratios that are consistent (i.e. > 1.4).

|  | **Avg. Abundance** | |  |  |
| --- | --- | --- | --- | --- |
| **Metabolic Processes** | **Hydrocarbon- impacted** | **Agricultural- impacted** | **Diss/**  **SD** | **Cum %** |
| Cofactors, Vitamins, Prosthetic Groups, Pigments | 0.08 | **0.19** | **1.67** | 12.09 |
| Virulence, Disease and Defence | 0.08 | **0.19** | **1.67** | 24.19 |
| Phages, Prophages, Transposable elements, Plasmids | 0.08 | **0.19** | **1.67** | 36.28 |
| Fatty Acids, Lipids, and Isoprenoids | 0.08 | **0.19** | **1.67** | 48.38 |
| Iron acquisition and metabolism | **0.84** | 0.79 | **1.76** | 54.29 |
| Dormancy and Sporulation | **0.71** | 0.67 | **1.4** | 58.92 |
| Metabolism of Aromatic Compounds | **0.87** | 0.84 | **1.82** | 62.37 |
| Photosynthesis | 0.69 | 0.69 | 1.39 | 65.57 |
| Motility and Chemotaxis | **0.83** | 0.8 | **1.67** | 71.84 |
| Protein Metabolism | 0.93 | **0.96** | **3.27** | 74.59 |
| Carbohydrates | 0.97 | **0.99** | **3.44** | 77.27 |
| Potassium metabolism | 0.79 | 0.78 | 0.82 | 79.84 |
| Nitrogen Metabolism | **0.84** | 0.81 | **1.84** | 82.37 |
| Regulation and Cell signalling | **0.85** | 0.83 | **1.81** | 84.78 |
| Amino Acids and Derivatives | 0.96 | **0.98** | **2.35** | 86.73 |
| Clustering-based subsystems | 0.97 | **0.99** | **1.75** | 88.4 |
| Miscellaneous | 0.94 | **0.95** | **2.42** | 89.89 |
| Respiration | 0.89 | **0.9** | **1.55** | 91.17 |
| Phosphorus Metabolism | **0.84** | 0.83 | **1.42** | 92.42 |
| RNA Metabolism | **0.92** | 0.83 | **1.77** | 93.65 |
| Cell Division and Cell Cycle | 0.84 | 0.84 | 0.67 | 94.86 |
| Sulfur Metabolism | 0.83 | 0.83 | **1.52** | 96.02 |
| Nucleosides and Nucleotides | 0.88 | **0.89** | **1.79** | 97.05 |
| Stress Response | 0.88 | 0.88 | 0.87 | 97.94 |
| Cell Wall and Capsule | 0.91 | 0.92 | 1 | 98.74 |
| DNA Metabolism | 0.91 | 0.91 | 1.01 | 99.53 |
| Membrane Transport | **0.89** | 0.88 | **1.46** | 100 |

Diss=dissimilarity; SD=Standard Deviation; Cum %=cumulative percentage of contribution to overall dissimilarity, Avg. Abundance values are reported for square-root transformed data

**Table S4 -** **Comparison of four separate CAP analyses comparing different hypotheses as discriminant functions within the same 14 metagenomic samples (see Table S1 for provenance).**

| **Hypothesis:** | **Contaminants** | **Habitat types** | **Geographic locations** | **Wet vs dry environments** |
| --- | --- | --- | --- | --- |
| **Variable** |  |  |  |  |
| **# groups compared** | 4 | 5 | 2 | 2 |
| **PC axes chosen *m*** | 2 | 5 | 1 | 1 |
| **Proportion of variation explained** | 88.6 | 97.7 | 80.2 | 80.2 |
| **Significance of trace statistic** | 0.0005  *** | 0.0016  ** | 0.0015  ** | 0.43  NS |
| **Significance of delta statistic** | 0.0005  *** | 0.0348  * | 0.0015  ** | 0.43  NS |
| **Total % of samples misclassified:** | 21.4 | 42.9 | 14.29 | 28.6 |
| **# samples correctly classified out of total** | hydrocarbon: 3/4  agriculture: 7/7  pristine: 0/1  wastewater: 1/2 | beach sand: 0/3  soil: 1/3  sediment: 4/4 groundwater: 1/2  wastewater: 2/2 | Southern Hemisphere: 9/10  Northern Hemisphere: 3/4 | wet: 4/6  dry: 6/8 |

**References**

68. Adetutu EM, Smith RJ, Weber J, Aleer S, Mitchell JG, et al. (2013) A polyphastic approach for assessing the suitability of bioremediation for the treatment of hydrocarbon-impacted soil

69. Albertsen M, Hansen LBS, Saunders AM, Nielsen PH, Nielsen KL (2012) A metagenome of a full-scale microbial community carrying out enhanced biological phosphorus removal. ISME J 6: 1094 - 1106.
